# A feasibility study into adenosine triphosphate measurement in exhaled breath condensate: a potential bedside method to monitor alveolar deformation

**DOI:** 10.1007/s11302-018-9607-6

**Published:** 2018-05-12

**Authors:** Philip van der Zee, Peter Somhorst, Jeroen Molinger, Djo Hasan, Diederik Gommers

**Affiliations:** 1 Department of Adult Intensive Care Medicine, Erasmus MC, Erasmus University Rotterdam, ‘s Gravendijkwal 230, 3015 CE Rotterdam, The Netherlands; 2 BeLife Human Performance Lab, Max Euwelaan 72, 3062 MA Rotterdam, The Netherlands; 3 Department of Surgery, Erasmus MC, Erasmus University Rotterdam, ‘s Gravendijkwal 230, 3015 CE Rotterdam, The Netherlands

**Keywords:** Adenosine triphosphate (ATP), Exhaled breath condensate (EBC), Exercise test, Luciferin-luciferase assay

## Abstract

Recent research suggested an important role for pulmonary extracellular adenosine triphosphate (ATP) in the development of ventilation-induced lung injury. This injury is induced by mechanical deformation of alveolar epithelial cells, which in turn release ATP to the extracellular space. Measuring extracellular ATP in exhaled breath condensate (EBC) may be a non-invasive biomarker for alveolar deformation. Here, we study the feasibility of bedside ATP measurement in EBC. We measured ATP levels in EBC in ten subjects before and after an exercise test, which increases respiratory parameters and alveolar deformation. EBC lactate concentrations were measured as a dilution marker. We found a significant increase in ATP levels in EBC (before 73 RLU [IQR 50–209] versus after 112 RLU [IQR 86–203]; *p* value 0.047), and the EBC ATP-to-EBC lactate ratio increased as well (*p* value 0.037). We present evidence that bedside measurement of ATP in EBC is feasible and that ATP levels in EBC increase after exercise. Future research should measure ATP levels in EBC during mechanical ventilation as a potential biomarker for alveolar deformation.

## Introduction

Recently, we suggested an important role for pulmonary extracellular adenosine triphosphate (ATP) in the development of ventilation-induced lung injury or acute respiratory distress syndrome (ARDS) [1]. However, at this moment, there is no clinically applicable method to detect extracellular ATP in the lungs.

ATP is omnipresent in cell tissues and the majority of ATP is located in the intracellular space [2–4]. Cells can release ATP molecules after a variety of stimuli (e.g., mechanical deformation, inflammation) and the extracellular ATP concentration increases [3]. In the lungs, stretch of the alveolar epithelial type I (AT I) cells results in the extracellular release of ATP [5–7]. Real-time imaging demonstrated that extracellular ATP release occurs simultaneously with mechanical deformation [8]. A nanomolar increase in extracellular ATP stimulates the alveolar epithelial type II cells to release surfactant in the alveolar space [7, 9–11]. Subsequently, extracellular ATP is converted by the CD39 and CD73 enzymes to adenosine and inosine [3, 4].

The amount of extracellular ATP release correlates with the magnitude of alveolar deformation [6]. Mechanical ventilation can induce severe mechanical deformation and subsequent massive ATP release into the extracellular space. Millimolar concentrations of extracellular ATP act as a danger-associated molecular pattern and initiate the pro-inflammatory innate immune response [3, 12, 13]. Prolonged exposure to high levels of extracellular ATP can result in ventilation-induced lung injury or ARDS [1]. The measurement of extracellular ATP in the lungs might be a biomarker for alveolar deformation.

ATP in the expired breath can be detected in exhaled breath condensate (EBC) [14–16]. EBC is collected by leading exhaled breath air from a subject through a thermo-electric cooling module. The resultant condensate is used for further analyses. EBC collection is a non-invasive method to acquire samples from the respiratory tract and alveoli [17–19]. It is a safe method to assess inflammatory biomarkers in various pulmonary diseases [20]. In addition, EBC contains only few cellular components and low protein levels, indicating virtually no ATP release and low conversion rate [14]. ATP has proven to be stable in EBC for at least 30 min [16]. Previous studies used a luciferin-luciferase assay to detect extracellular ATP [14–16], a highly sensitive method to detect ATP [21]. In this study, we used a handheld luminometer with a ready to use assay kit. This allowed us to perform ATP measurements in a bedside manner.

We collected EBC from subjects before and after exercise to test whether bedside ATP measurements were feasible. Exercise results in a wide range of physiologic responses, including a significant increase in respiratory parameters (e.g., tidal volume, respiratory rate, and respiratory minute volume) [22]. We hypothesized that the increase in respiratory parameters during exercise resulted in a rise in alveolar deformation and subsequent ATP release into the extracellular space. The aim of this study was to assess the feasibility of bedside ATP measurements and to measure ATP levels in EBC before and after an exercise test.

## Methods

### Study design and setting

This prospective observational study was performed at BeLife Human Performance Lab, a performance screen and rehabilitation center. We included subjects between 18 and 75 years old who had a cycle ergometry exercise test at BeLife between October 2017 and January 2018. The exclusion criteria were age < 18 years, new onset respiratory symptoms in the past week, and/or a history of unstable respiratory disease (asthma, chronic obstructive pulmonary disease, interstitial lung disease, or pulmonary malignancy) requiring changes in therapy in the past 3 months. The primary outcome of this study was the difference in ATP levels in EBC before and after an exercise test. In addition, ATP levels in EBC were correlated with the following respiratory parameters: respiratory rate, tidal volume, and respiratory minute volume. This study was commissioned by the Department of Adult Intensive Care Medicine of the Erasmus MC Rotterdam, the Netherlands. The study has been performed in accordance with the 1964 Declaration of Helsinki and its later amendments. All subjects gave written informed consent.

### Data collection

#### Cycle ergometry exercise test

All subjects performed a cycle ergometry exercise test according to the local ramp protocol. The test consisted of a gradual increase in workload until exhaustion. Hemodynamic, metabolic, and respiratory parameters, including respiratory rate, tidal volume, and respiratory minute volume, were recorded. Measurement of height and weight and spirometry (Jaeger Vyntus CPX, Vyaire Medical, USA) were performed before the exercise test. Before and after exercise, a capillary blood gas sample was taken. If a capillary blood gas sample after exercise could not be obtained, blood lactate was measured using Lactate Pro2 LT-1730 (Arkray, Japan).

#### Exhaled breath condensate

EBC was collected with the commercially available TurboDECCS System exhaled breath condensator (Disposable Exhaled Condensate Collection Systems, DECCS, Medivac, Italy). A disposable TurboDECCS mouthpiece with saliva filter designed for spontaneously breathing subjects was used. We set condensation temperature at − 7 °C. EBC was collected twice: once directly before and once 5 min after the exercise test. Subjects exhaled through the mouthpiece during 15 min of tidal breathing. EBC was collected during 15 min to collect sufficient sample volume; duration of EBC sampling does not influence adenosine concentrations [17]. In order to minimize sensations of shortness of breath or faintness after the exercise test, no nose clip was required.

#### Luciferin-luciferase assay

ATP levels in EBC were measured with luminometry and luciferin-luciferase assay. In this study, a 3-M ready to use luciferin-luciferase water assay kit (3 M Clean-Trace Luminometer LM1, Neuss, Germany) was used. The amount of ATP was expressed in relative light units (RLU). The linearity and sensitivity of this luminometer was confirmed by measurements with different concentrations of sterile pure ATP solutions ranging from 10^−11^ to 10^−5^ M [23]. These ATP concentrations corresponded with 10^1^ to 10^6^ RLU. Two hundred microliters of EBC was pipetted directly into each assay kit using disposable pipette tips (Filter tip, Greiner Bio-one, Austria). The assay was repeatedly performed every 15 s for a duration of 2 min until an equilibrium was reached, i.e., stable RLU values during at least two measurements. In order to decrease intra-assay variability, the luciferin-luciferase assay was repeated three times with different assay kits for every EBC sample. Mean ATP level of the three equilibrium values was used in the analyses and intra-assay coefficient of variation (CV) was calculated.

#### Dilution marker and amylase assay

We used EBC lactate as a marker for EBC sample dilution and calculated EBC ATP-to-EBC lactate ratio. In one occasion, insufficient sample material was collected and median EBC lactate was used. Lactate in capillary blood gas and EBC was performed on a RapidPoint 500 System (Siemens, Germany, detection limit 180 μmol/L). Subsequently, EBC was stored at − 80 °C for amylase assay. A colorimetric (405 nm) amylase assay was performed to detect possible saliva contamination. Amylase activity was assessed using an Amylase Activity Assay Kit (MAK009, Sigma-Aldrich, USA) and a Varioskan LUX multimode microplate reader (Thermo Fischer Scientific, USA) according to manufacturer protocol.

### Sample size and statistical analysis

We did not calculate a sample size, as the change in ATP levels in EBC before and after exercise is currently unknown. We decided to include ten subjects in this feasibility study. Baseline characteristics and exercise test data were presented as descriptive statistics. Data was tested for normality. As most data was not normally distributed, continuous data were reported as median and interquartile range (IQR). A related samples Wilcoxon signed rank test was used to assess differences before and after the exercise test. All statistical analyses were performed in IBM SPSS Statistics 21. A *p* value < 0.05 was considered statistically significant.

## Results

### Subject characteristics before and after the exercise test

Twelve subjects were enrolled in this study. One EBC sample obtained before the exercise test contained substantial traces of amylase, while the other samples had an absorbance similar to background signal. We considered this sample to be contaminated with saliva and the subject was excluded from analyses. Another subject was excluded as no EBC was collected despite multiple attempts. The characteristics of the ten included subjects are presented in Table 1. Only two subjects had no medical history, as BeLife is both a performance screen center and a rehabilitation center. The results of the exercise tests are shown in Table 2. Both hemodynamic and metabolic parameters increased significantly during exercise. Respiratory parameters, including respiratory minute volume, increased significantly as well. This was also reflected in a statistically significant decrease in pCO_2_ after the exercise test. In the capillary blood gas, there was a significant change in HCO_3_^−^, base excess, and lactate.

**Table 1:** Demographic and clinical characteristics of the subjects (*n* = 10)

Characteristic	Median	IQR
Female	*n* = 8 (80%)	
Age (years)	46	30–53
Height (cm)	170	166–177
Weight (kg)	73.1	61.2–95.2
BMI	26.0	22.1–32.0
BSA (m^2^)	1.90	1.74–2.10
Duration of exercise test (min:s)	9:46	7:11–11:08
Medical history	Obesity (*n* = 3) Asthma (*n* = 1) Surgery (*n* = 1) Intensive care admission (*n* = 1) Essential thrombocytosis (*n* = 1) M. Crohn (*n* = 1) No medical history (*n* = 2)	
Current smoking	*n* = 1	
Recent respiratory symptoms	*n* = 2	
Spirometry		
Forced vital capacity (L)	4.00	3.01–4.54
FEV_1_ (L)	3.09	2.32–3.89
FEV_1_ predicted (%)	100	90–111
FEV_1_/VC (%)	80.6	73.7–84.5

*BMI* body mass index, *BSA* body surface area, *FEV*_*1*_ forced expiratory volume in one second, *IQR* interquartile range, *VC* vital capacity

**Table 2:** Physiologic variables before and after the exercise test

Variables	Unit	Before exercise (rest)		After exercise (peak VO_2_)		*p* value
Hemodynamic parameters						
Heart rate	1/min	87	(74–97)	172	(147–189)	< 0.01*
Systolic blood pressure	mmHg	129	(125–158)	185	(167–213)	< 0.01*
Diastolic blood pressure	mmHg	77	(68–93)	79	(74–94)	0.959
MAP	mmHg	94	(89–117)	112	(106–131)	< 0.01*
Metabolic parameters						
VO_2_	mL/min	345	(297–413)	2047	(1599–2436)	< 0.01*
Respiratory exchange ratio		0.78	(0.72–0.89)	1.10	(1.03–1.27)	0.014*
PETCO_2_	mmHg	33.75	(27.76–36.25)	33.28	(29.42–38.21)	0.721
EqCO_2_		33.0	(30.3–35.7)	33.6	(27.7–36.8)	0.959
MET		1.1	(1.0–1.6)	7.7	(6.1–10.9)	< 0.01*
Respiratory parameters						
Tidal volume	L	0.744	(0.533–0.883)	2.261	(1.809–2.652)	< 0.01*
Respiratory rate	1/min	15.4	(12.7–17.2)	40.1	(31.5–44.1)	< 0.01*
Respiratory minute volume	L/min	11.5	(8.7–13.4)	87.1	(64.4–112.3)	< 0.01*
Capillary blood gas						
pH		7.408	(7.398–7.442)	7.358	(7.290–7.387)	0.080
pCO_2_	mmHg	35.0	(30.2–35.9)	31.2	(27.1–33.9)	0.042*
pO_2_	mmHg	75.2	(62.1–85.0)	91.5	(90.9–97.4)	0.068
HCO_3_^−^	mmol/L	21.5	(21.0–22.1)	15.1	(13.7–19.8)	0.043*
Base excess		− 2.1	(− 3.2; − 1.6)	− 9.7	(− 10.9; − 4.3)	0.043*
Hematocrit	mmol/L	0.41	(0.35–0.43)	0.43	(0.40–0.46)	0.102
Hemoglobin	mmol/L	8.6	(7.4–9.1)	9.1	(8.3–9.7)	0.066
Oxygen saturation		0.95	(0.92–0.96)	0.96	(0.96–0.98)	0.068
Lactate	mmol/L	1.63	(1.32–1.83)	7.82	(5.63–9.79)	0.018*

Data are presented as median and interquartile range unless stated otherwise

*VO*_*2*_ volume of oxygen consumption, *MAP* mean arterial pressure, *PETCO*_*2*_ partial pressure of exhaled carbon dioxide, *MET* metabolic equivalent of a task

*p value < 0.05

### ATP in exhaled breath condensate

**Fig. 1:**
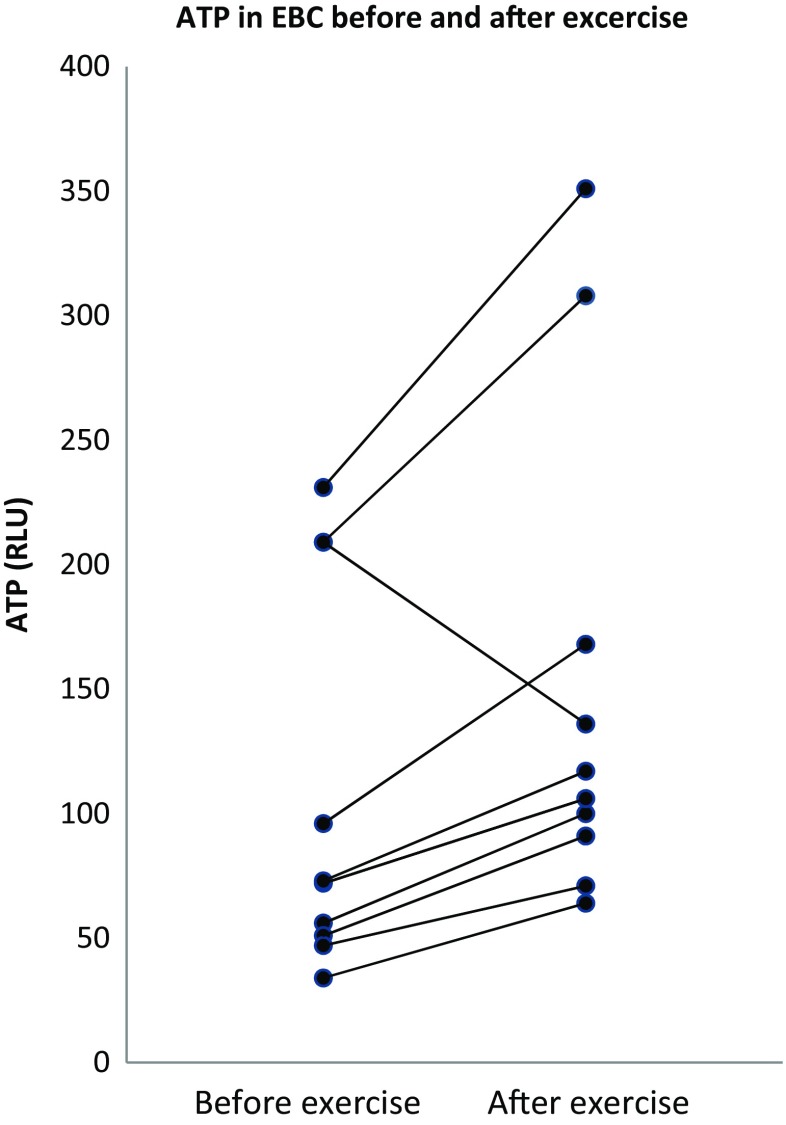
Adenosine triphosphate in exhaled breath condensate before and after exercise

The ATP levels in EBC were detectable in all subjects and increased in nine out of ten subjects (Fig. 1). ATP levels in EBC increased significantly after exercise (112 RLU, [IQR 86–203]) as compared to before the exercise test (73 RLU, [IQR 50–209]; *p* value 0.047) (Table 3). Lactate concentrations measured in EBC as a dilution marker did not differ before and after exercise. Comparison of EBC ATP-to-EBC lactate ratio before and after the exercise test resulted in a significant increase (*p* value 0.037) as well. The ATP measurements were reproducible with an intra-assay CV of 9.8%. Collected EBC volume was significantly greater after the exercise test, while collection time was similar. No adverse events were observed during this study. We did not find a significant linear correlation between respiratory rate, tidal volume, or respiratory minute volume and the amount of ATP detected.

**Table 3:** Adenosine triphosphate in exhaled breath condensate (EBC)

Exhaled breath condensate	Unit	Before exercise (rest)		After exercise (peak VO_2_)		*p* value
EBC ATP	RLU	73	(50–209, range 34–231)	112	(86–203, range 64–351)	0.047*
EBC lactate	mmol/L	0.44	(0.41–0.48)	0.45	(0.42–0.49)	0.573
EBC ATP-to-EBC lactate ratio		176	(109–444, range 78–525)	278	(186–486, range 131–780)	0.037*
Time of EBC collection	min:s	15:00	(14:48–15:00)	15:00	(14:48–15:00)	0.317
EBC volume	mL	1.3	(0.8–2.0)	1.9	(1.2–2.1)	0.038*

Data are presented as median and interquartile range unless stated otherwise

*RLU* relative light units

*p value < 0.05

## Discussion

This study showed that the bedside measurement of ATP levels in EBC is feasible. We found a significant increase in ATP levels in EBC after the exercise test as compared to before exercise. Lactate concentrations in EBC, measured as a dilution marker, were similar before and after the exercise test. In addition, we confirmed that EBC collection is simple and safe.

ATP levels in EBC increased in nine out of ten subjects after exercise. Although exercise induces multiple systemic responses, as indicated by a significant increase in physiologic parameters and especially blood lactate concentration, we hypothesized that increased alveolar deformation is the main reason for the observed increase in ATP levels. A systemic origin of increased ATP levels in EBC after exercise is unlikely, as extracellular ATP is rapidly degraded by both soluble and membrane-bound ecto-enzymes [3]. In addition, in healthy lungs, the tight junctions between adjacent pulmonary epithelium seal the cells and form a barrier between the alveolar air space and the interstitium [24, 25]. Barrier function can diminish following cell damage or inflammation, but it remains intact during brief exercise. This is also reflected by the fact that blood lactate concentration increased significantly, while EBC lactate concentration remained unchanged. As lactate (89 g/mol) is a significantly smaller molecule than ATP (507 g/mol) [26], a rise in lactate concentration in EBC through paracellular transport is more likely to occur. Thus, it is possible that the lung itself is the source of increased ATP levels in EBC after exercise. In one subject, ATP levels in EBC did not increase after exercise. This subject was stressed before the exercise test, as indicated by highest heart rate and respiratory parameters at rest. She was in excellent physical condition and recovered fast with a heart rate below baseline at 120 s after exercise. Therefore, the difference in physiologic variables before and after the exercise test was smallest in this subject. Other possible explanations for the decrease in ATP levels are contamination of the sample (other than saliva) acquired before the exercise test, or increased instability of ATP after the exercise test due to a change in EBC composition after exercise (e.g., pH) [27, 28].

### EBC composition and origin

In theory, EBC originates from the entire respiratory tract, although the exact origin of EBC remains unclear [29, 30]. The composition of EBC corresponds with the composition of airway lining fluid (ALF) [29], although solute concentrations are significantly lower. EBC is generated in a milieu of air that is nearly saturated with gas-phase water vapor; the majority of EBC consists of evaporated water (up to 99.9%) [17, 31–33]. The remainder EBC fluid contains a multitude of volatile and non-volatile compounds. The non-volatile compounds in ALF undergo aerosolization during tidal breathing as small droplets of ALF are released from the airway surfaces [29, 30, 32]. The number of particles detected in exhaled air varies between 0.1 and 4.0 particles per milliliter [34]. Multiple models have been proposed to explain particle aerosolization, including airway turbulence, thermodynamic aerosol formation, and the bronchiole fluid film burst (BFFB) model [30, 35, 36]. Airway turbulence, however, is an improbable source of aerosolization in EBC as flow is laminar in the bronchiole at naturally achieved flow rates [35].

Several studies assessed the influence of exercise on the composition of EBC. The majority of ions and compounds remained unchanged [27], although a significant increase in EBC pH was reported [27, 28]. Both unchanged and increased lactate concentrations in EBC after exercise were observed [27, 37]. The EBC lactate concentrations in this study were in concordance with previously measured concentrations [38]. ATP concentrations in EBC have been measured in patients with COPD, asthma, and cystic fibrosis. These studies reported some variability in ATP concentrations [14–16]. However, they did demonstrate a decrease in ATP levels after antibiotic treatment of pulmonary cystic fibrosis exacerbations [16].

Limitations from this study mainly derived from the low particle concentrations found in EBC and the absence of EBC collection and sample handling standardization. The largest pitfall of analyses of EBC is the unknown amount of fragmented droplet aerosols. According to the BFFB model, an increase in respiratory minute volume should lead to an increased number of expired particles [39]. This does not significantly influence EBC sample dilution, as the total amount of exhaled water increases as well [40]. Nevertheless, our subjects had to recover at least 5 min in order to partially restore normal respiratory minute volume. Previous studies reported a wide range in EBC adenosine concentrations and calculated a purine-to-urea ratio to correct for dilution variability [41–43]. Significant amounts of urea and lactate have been observed in EBC [38]. In theory, both can be used as a denominator for the unknown amount of particles that has been aerosolized. Previously, urea was used as it is not produced or metabolized in the lungs [44, 45], despite a great within-subject variability in EBC urea concentrations [17, 46]. In our study, lactate concentrations were comparable before and after exercise, although lactate can be produced by the respiratory epithelium [27]. As EBC lactate can increase during exercise, an EBC ATP-to-EBC lactate ratio might underestimate the true increase in ATP levels. ATP levels in EBC are near the lower detection limit with the bedside luminometer used in this study. Intra-assay variability was 9.8% despite low ATP levels in EBC; a CV of 10% is considered acceptable [47]. The CV tended to decline as ATP levels in EBC were greater. Increasing the lower detection limit would not only increase test sensitivity, but decrease test variability in the lower ranges as well. According to previously published calibration curves, we estimate that EBC ATP levels in our study were in nanomolar ranges [23]. Although ATP levels measured in EBC are underestimated; a part of extracellular ATP is rapidly converted to adenosine [3]. Despite supervised EBC collection and saliva filter in the TurboDECCS mouthpiece, one sample was tested positive for amylase. According to literature, sample contamination rarely occurs and routine amylase assay is not recommended [17, 33]. However, sample contamination is unacceptable when purine concentrations are measured. Therefore, we recommend routine amylase assay in EBC collection of spontaneously breathing subjects. We did not estimate a sample size to detect a correlation between respiratory parameters and an increase in ATP levels. Moreover, substantial variability between subjects obscured any correlation. Because of the great variability in exhaled aerosol concentrations between subjects, longitudinal measurements and intra-individual comparisons are preferable [48]. In addition, the within-subject change in ATP levels was assessed, as reference values for inflammatory biomarkers in EBC remain to be established [18, 29, 49].

## Conclusions

In the present study, we confirmed that it is feasible to measure ATP levels in EBC in a bedside manner. In addition, ATP levels in EBC increased after exercise, whereas lactate concentrations in EBC remained similar. We hypothesized that ATP levels increased as a result of alveolar deformation. Although EBC collection has some pitfalls and may underestimate alveolar extracellular release of ATP, the non-invasive measurement of ATP levels in EBC holds great potential. Measurement of ATP in EBC may provide a relatively simple and non-invasive method to monitor alveolar deformation. Future studies will focus on the measurement of ATP in EBC during mechanical ventilation.
